# The Reasons for Excess Mortality during the COVID-19 Pandemic

**DOI:** 10.4269/ajtmh.22-0243b

**Published:** 2022-06-13

**Authors:** Christopher T. Leffler, Sneha Konda

**Affiliations:** Department of Ophthalmology Virginia Commonwealth University Richmond, Virginia, and Department of Ophthalmology Hunter Holmes McGuire VA Medical Center Richmond, Virginia E-mails: chrislefflermd@gmail.com and sneha.konda@vcuhealth.org

Dear Sir,

We agree with Drs. Mattiuzzi and Lippi that excess mortality during the pandemic is not exactly equivalent with mortality caused by SARS-CoV-2 infection.^1^ We noted that excess mortality may result from “Health system overload, delays in patients seeking unrelated healthcare … social changes such as lockdowns … other diseases, war, or environmental factors. Mortality deficits … may result from fewer injuries.”^2^

We found that the excess mortality during the COVID-19 pandemic in India, through August 31, 2021, was 198.7 per 100,000 population (range 146.1–263.8 per 100,000).^2^ For context, the Hopkins dataset we used reflected COVID-19 mortality of 203.1 per 100,000 population in the United States as of August 31, 2021.^2^ So, the excess mortality in India is comparable with the COVID-19 mortality in countries with more robust testing. The survey that Drs. Mattiuzzi and Lippi cited does not demonstrate that delays in seeking healthcare resulted in increased mortality of this magnitude.^3^ The survey respondents obviously had not died, and the survey did not ask whether anyone in the family had died as a result of delayed care.^3^

The most disruptive lockdowns in India occurred in Spring 2020.^4^ However, the bulk of the excess mortality in India occurred in Spring 2021, coincident with the delta-variant COVID-19 wave, and to a lesser degree in Fall 2020.^2^ Thus, we believe that the bulk of the excess mortality in India was in fact due to COVID-19 infection.
